# Epilepsy Associated With Mitochondrial Encephalomyopathy, Lactic Acidosis, and Stroke-Like Episodes

**DOI:** 10.3389/fneur.2021.675816

**Published:** 2021-06-11

**Authors:** Jiaai Li, Wuqiong Zhang, Zhitao Cui, Zhaoran Li, Ting Jiang, Hongmei Meng

**Affiliations:** ^1^Department of Neurology and Neuroscience Center, The First Hospital of Jilin University, Changchun, China; ^2^Department of Geriatrics, The First Hospital of Jilin University, Changchun, China

**Keywords:** MELAS, epilepsy, prognosis, influencing factors, EEG

## Abstract

**Objectives:** The present study explored the clinical characteristics and prognostic factors of epilepsy in patients with mitochondrial encephalomyopathy, lactic acidosis, and stroke-like episodes (MELAS).

**Methods:** Thirty-four MELAS patients were included in the present study. They were diagnosed by clinical characteristics, genetic testing, muscle biopsy, and retrospective analysis of other clinical data. The patients were divided into three groups according to the effects of treatment after at least 2 years of follow-up.

**Results:** Epilepsy was more common in male MELAS patients than in females (20/14). The age of onset ranged from 0.5 to 57 years, with an average of 22.6 years. Patients with epilepsy and MELAS had various forms of seizures. Focal seizures were the most common type affecting 58.82% of patients, and some patients had multiple types of seizures. The abnormal EEG waves were mainly concentrated in the occipital (69.57%), frontal (65.22%) and temporal lobes (47.83%). Overall, the prognosis of patients with epilepsy and MELAS was poor. Poor prognosis was associated with brain atrophy (*P* = 0.026), status epilepticus (*P* < 0.001), and use of anti-seizure medications with high mitochondrial toxicity (*P* = 0.015).

**Interpretation:** Avoiding the application of anti-seizure medications with high mitochondrial toxicity, controlling seizures more actively and effectively, and delaying the occurrence and progression of brain atrophy as much as possible are particularly important to improve the prognosis of patients with MELAS and epilepsy.

## Introduction

Mitochondrial diseases cause mitochondrial dysfunction due to abnormalities in mitochondrial DNA (mtDNA) and/or nuclear DNA (nDNA) which eventually leads to a series of clinical symptoms affecting the muscle and central nervous symptoms. The clinical manifestations of mitochondrial encephalomyopathy are diverse, and its prevalence is ~1/5,000 (1). MELAS is the most common mitochondrial encephalomyopathy and follows a pattern of maternal inheritance. When only muscles are affected, the disease is referred to as mitochondrial myopathy, and when the central nervous system or other systems are also affected, it is referred to as mitochondrial encephalomyopathy. In clinical practice, mitochondrial encelphalomyopathy is more common than mitochondrial myopathy. Mitochondrial encephalomyopathy is one of the most common forms of hereditary and metabolic neuromuscular diseases, and mitochondrial encephalopathy with lactic acidosis and stroke-like episodes (MELAS) is one of the most common maternally inherited mitochondrial disorders (2). MELAS is a well-defined clinical syndrome that is characterized by recurrent stroke-like seizures, epilepsy and headaches (3), as well as dementia, hyperlacticemia, myopathy, hearing impairment, diabetes and short stature.

Epilepsy is very common in MELAS patients, and it is reported that 71–96% of MELAS patients experience seizures (4). The mechanism of the epilepsy associated with MELAS is not fully understood. Current widely accepted mechanisms include oxidative stress damage, ion mechanisms, energy mechanisms and neurotransmitter mechanisms (5, 6). Repeated occurrence of seizures aggravates mitochondrial damage, therefore, it is generally believed that there is a vicious cycle of mitochondrial damage and seizures in MELAS patients (7–9). The epilepsy associated with MELAS may develop into non-convulsive or convulsive status epilepticus, which seriously affects prognosis. Effective control of seizures in MELAS patients can slow brain tissue metabolism, reduce lactic acid accumulation and even lower the risk of stroke-like seizures. However, the nature of mitochondrial dysfunction makes the epilepsy associated with MELAS difficult to treat, and there are limited options for clear and effective drugs (6). Therefore, understanding the clinical characteristics and prognostic factors of patients with epilepsy associated with MELAS can enable clinicians to better manage these patients' conditions and improve their quality of life.

This study explored the clinical characteristics and prognostic factors of epilepsy in patients with mitochondrial encephalomyopathy, lactic acidosis, and stroke-like episodes (MELAS). It also provides a basis for the clinical diagnosis, treatment, and prognosis of these patients.

## Materials and Methods

Excluding cases of epilepsy attributed to other causes, a total of 34 patients with MELAS (aged 0.5–57 years, 20 males and 14 females) were evaluated. The patients were admitted to First Hospital of Jilin University, China, from 2000 through 2018. All patients had a confirmed diagnosis of MELAS and epilepsy based on the diagnostic criteria reported by Yatsuga et al. (10) as well as the ILAE 2017 classification of seizure types (11) and by clinical characteristics, muscle biopsy, genetic testing, and video electroencephalogram. Following confirmation of diagnosis, these patients underwent a more detailed assessment. Laboratory test results (cardiac function marker, liver function, renal function, resting venous lactic acid), clinical data, electroencephalography (EEG) and imaging were obtained during a follow-up period of at least 2 years. Each patient received anti-epileptic medication after the diagnosis of epilepsy was confirmed. We divided the subjects into three groups based on the occurrence of seizures after at least 2 years of follow-up: complete control group (*n* = 7), effective group (*n* = 12) and the ineffective group (*n* = 15). The complete control group included patients for whom the number of seizures was reduced by 100% compared to the baseline. The effective group included patients for whom the number of seizures was reduced by 50–75%; all other patients were in the ineffective group. We obtained informed consent from the patients and families to obtain the patients clinical data.

All analyses were performed using SPSS version 24.0. Descriptive statistics, including the mean, standard deviation, median, and range, were determined. The prognostic factors of epilepsy associated with MELAS were evaluated using Fisher's exact test and Mann-Whitney *U*-test. *P*-values <0.05 were considered statistically significant.

## Results

### Clinical Features of Subjects

**Figure 1:**
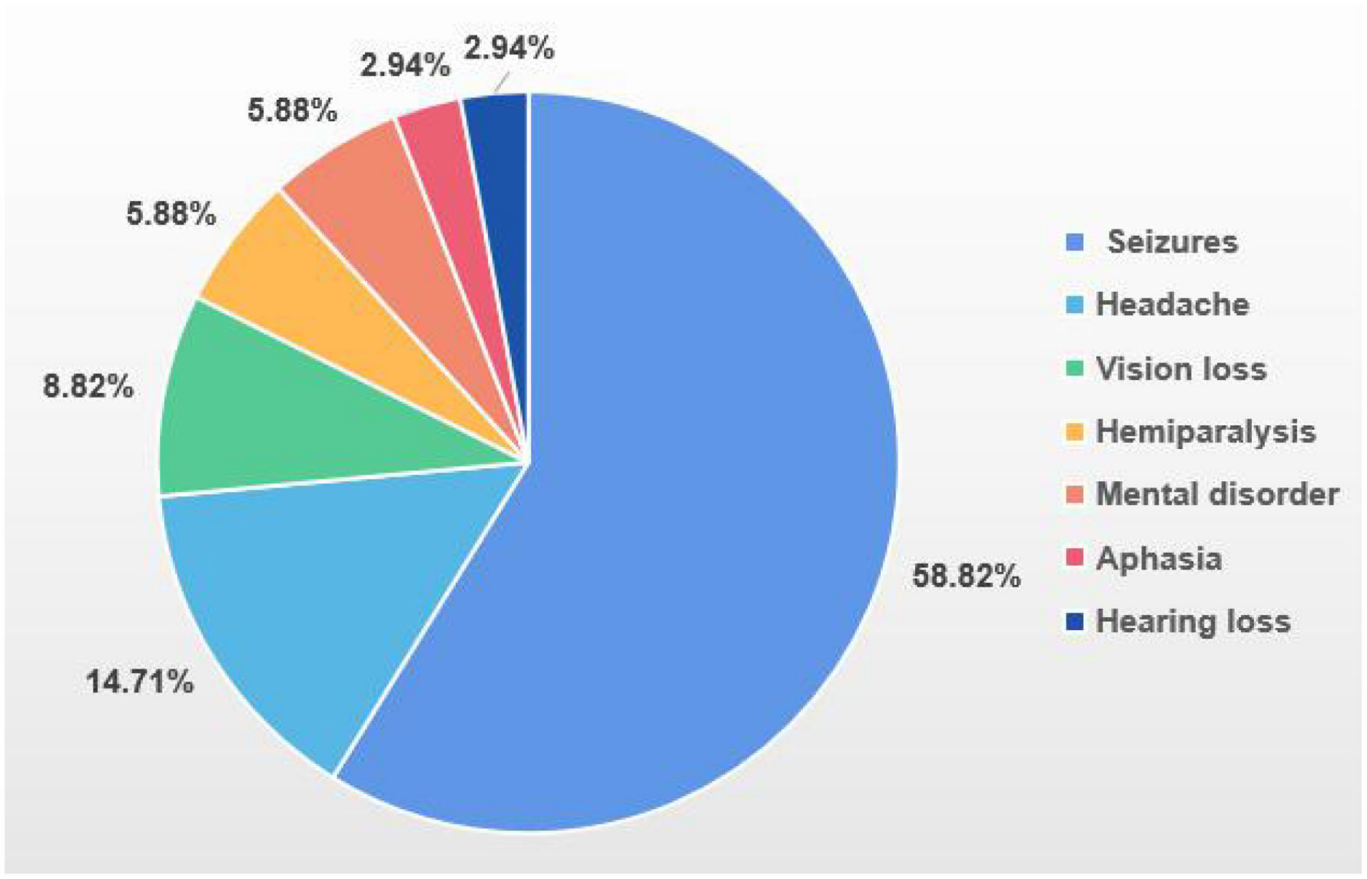
Initial neurological symptoms.

The mean age of the 34 patients at the first appearance of their symptoms was 22.6 years. The time between the appearance of epilepsy to a diagnosis of MELAS was 0–12 years. The initial neurological symptoms primarily included seizures (58.82%), headaches (14.71%) and vision loss (8.82%). Three patients also had nephropathy (8.82%) and three had diabetes (8.82%) (Figure 1). The 12 who underwent genetic testing for MELAS disease-related mutation hotspots had A3243G heterozygous point mutations in their mtDNA. Among the 24 patients who underwent skeletal muscle biopsy, 20 samples showed typical ragged-red fiber (RRF) after Gomori Trichrome staining, and four cases showed atypical RRF.

### Types of Seizures

**Figure 2:**
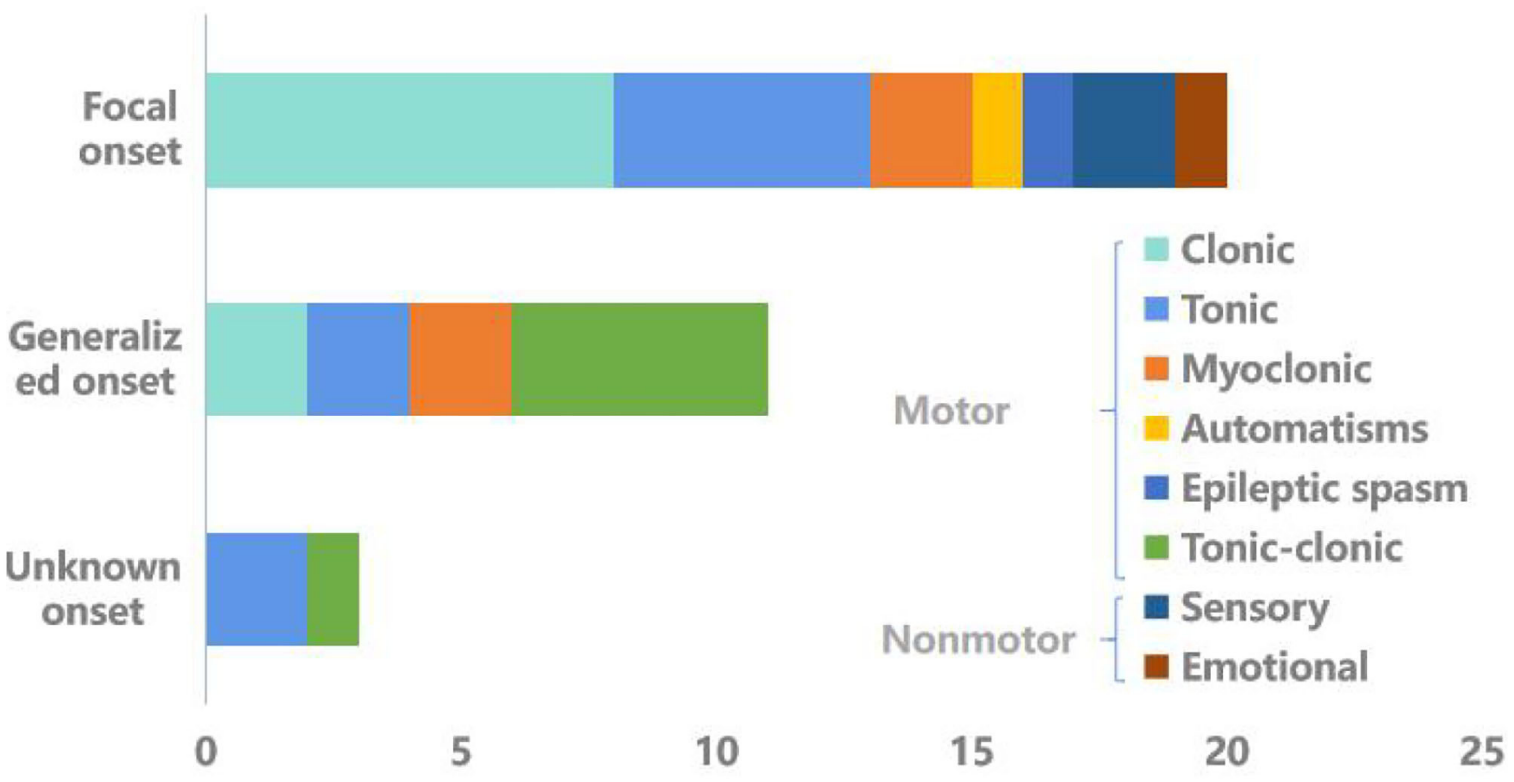
Types of seizures.

The characteristics of the seizure are listed in Figure 2. Focal onset was most common (58.82%), followed by generalized onset (32.35%). Three cases (8.82%) were of unknown onset. In the focal onset group, eight patients (23.53%) were aware and 12 (35.29%) had impaired awareness. Besides 35.29% of subjects had motor status epilepticus.

### Characteristics of the EEGs

**Figure 3:**
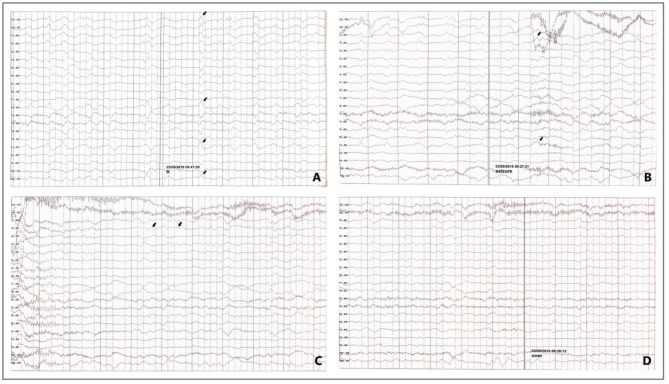
EEG in a patient with Epilepsy Associated with MELAS. Interictal stage: sharp waves are occasionally seen on the right side (**A**, arrows); Ictal stage: The patient felt abnormal paresthesias in the left hand. In the meantime, the EEG showed irregularly sharpened delta waves (**B**, arrows) in the background, and the amplitude gradually increased and evolved into 4–5 HZ slow activity (**C**, arrows), voltage recovery after 30–50 S **(D)** (focal onset, right central-temporal origin).

EEG abnormalities were seen in 91.3% of patients. The abnormal waveforms were mainly distributed in the occipital (16, 69.57%), frontal (15, 65.22%), temporal (11, 47.83%) and parietal (8, 34.78%) lobes. Of the patients, 43.48% had epileptiform discharges during epilepsy, which included general epileptiform discharges (21.74%) and focal epileptiform discharge (21.74%). 82.61% of patients who had interictal epileptiform discharges, mainly focal epileptiform discharges (69.57%). Three patients had extensive spikes and slow waves. Slow waves were seen in 13 patients (56.52%), mainly focal slow waves (10 cases, 43.48%). As shown in Figure 3, EEG of one patient revealed abnormal wave forms of interictal (Figure 3A, arrows) and ictal stage (Figures 3B–D, arrows).

### Manifestations on the Neuroimages

Among patients from whom a cranial MRI was obtained, 57.14% (16/28) of the lesions involved both the supratentorial and subtentorial regions, and 35.71% (10/28) involved only the supratentorial. The supratentorial lesions were mainly bilateral, only a few involving the left side (5, 17.86%) or the right side (3, 10.71%). The lesions were mainly distributed in the temporal (24, 85.71%), parietal (24, 85.71%) and occipital (21, 75%) lobes, and a few were distributed in the frontal lobe, basal ganglia, lateral ventricle, hippocampus, and insula. The subtentorial lesions were distributed in the cerebellum (11, 39.29%) and the brain stem (2, 7.14%). Of patients, 64.29% (18/28) had brain atrophy, and 32.14% (9/28) of them had atrophy in the brain and cerebellum. Of the patients from whom a brain CT scan was obtained, 26.67% (4/15) had brain tissue calcification, which was mainly concentrated in the basal ganglia (20%, 3/15). Eight patients underwent MRS spectroscopic imaging; of those, four had obvious lactate peaks, and three patients had decreased NAA peaks.

### Laboratory Examination

The following tests were performed on all patients at the initial stage of onset: cardiac function, liver function, renal function, resting venous lactate value. Of the patients, 44.12% had abnormal cardiac function; 23.53% had abnormal liver function; and 32.35% had abnormal renal function. As many as 81.25% (26/32) of patients had a high resting venous lactate value, and 83.33% (5/6) of patients had higher post-exercise venous blood lactate levels than at rest.

### Treatment and Prognosis

Most patients received the following symptomatic and supportive treatments: Coenzyme Q10 (23, 67.65%), high-dose vitamins (22, 64.71%), L-carnitine (12, 35.29%) and adenosine triphosphate (5, 14.71%). Seven patients (20.59%) received arginine treatment in the acute phase. All patients took anti-seizure medications (ASMs) after confirmation of their epilepsy diagnoses; 73.53% (25) underwent single-agent therapy, and 26.47% (9) underwent multi-drug therapy. Of the patients, 94.12% had good compliance with ASMs regimens. Among them, 47.06% (16) patients used ASMs with high mitochondrial toxicity, such as valproic acid, carbamazepine, phenytoin and phenobarbital. At follow-up after 2 years, 20.59% (7) of the patients had a 100% reduction in the number of seizures compared to the baseline, and they considered their epilepsy to be completely under control; 35.29% (12) of the patients had a 50–99% reduction in the number of seizures compared to the baseline, and the treatment was considered effective. Of the patients, 44.12% (15) had less than a 50% reduction in seizures compared to the baseline, and their treatments were considered ineffective. The rate of treatment effectiveness was 55.88% (Inefficiency = invalid/total number of patients× 100%).

Other clinical symptoms observed during the 2-year follow-up mainly included exercise intolerance and intellectual disability. Some symptoms improved to varying degrees, including mental symptoms, clumsy speech, loss of vision, paresthesia, headache, poor physical activity, and hearing loss. At the end of the follow-up period, only one of the 34 patients had died due to end-stage epilepsy and related complications (respiratory failure).

Statistical analysis of clinical data from patients in the complete control group, effective group and ineffective group, it is found that a higher proportion of patients in the ineffective group experienced status epilepticus (*P* < 0.001) and brain atrophy (*P* = 0.026) and used anti-epileptic drugs with excessive mitochondrial toxicity (*P* = 0.015), indicating that they are related to poor prognosis (Table 1).

**Table 1:** Prognostic factor.

**Variable**	**Onset age (<18)**	**Sex (male)**	**Types of Seizures**			**Status epilepticus**	**Brain atrophy**	**EEG**			**Treatment**		
			**Aware impaired (focal onset)**	**Focal onset (motor)**	**Generalized onset (motor)**			**Epileptic discharge**	**Slow wave**	**Background rhythm**	**Multi-ASMs**	**High mitochondrial toxicity ASMs**	**Poor adherence to ASMs**
Complete control group (*N* = 7)	2 (28.6)	6 (85.7)	1 (33.3)	2 (66.7)	2	0 (0.0)	1 (16.7)	1 (25.0)	1 (25.0)	2 (50.0)	1 (14.3)	3 (42.9)	1 (14.3)
Effective group (*N* = 12)	3 (25.0)	5 (41.7)	1 (14.3)	7 (100.0)	4	1 (8.3)	6 (75.0)	2 (28.6)	5 (71.4)	2 (28.6)	1 (8.3)	2 (16.7)	0 (0.0)
Ineffective group (*N* = 7)	8 (53.3)	9 (60.0)	6 (60.0)	8 (80.0)	5	11 (73.3)	11 (78.6)	7 (58.3)	7 (58.3)	6 (50.0)	6 (40.0)	11 (73.3)	1 (6.7)
*P*	0.281	0.178	0.177	0.348	…	<0.001^*^	0.026^*^	0.338	0.338	0.647	0.134	0.015^*^	0.447

*ASMs, anti-seizure medications; EEG, electroencephalography*.

* *and the bold mean that P-values less than 0.05 were considered statistically significant*.

## Discussion

Mitochondrial encephalomyopathy is a genetic disease in which defects in mtDNA or ntDNA cause abnormal oxidative phosphorylation of the mitochondrial respiratory chain, and this simultaneously affects the central nervous system and muscular system. The prevalence of mitochondrial encephalomyopathy is about 1/5,000, and the clinical manifestations are diverse. MELAS is the most common mitochondrial encephalomyopathy and follows a pattern of maternal inheritance. Epileptic seizures are the most common neurological symptom, and patients often experience multiple types of seizures (12). MELAS is metabolic epilepsy, and it can develop into status epilepticus. In keeping with other metabolic disorders, seizures include both spontaneous epileptic seizures and provoked seizures. The epileptic seizures in mitochondrial disorders differ from seizures in other contexts with respect (1) more frequently of posterior quadrant and occipital lobe onset; (2) more likely to present with non-convulsive status epilepticus which may last months; (3) multidrug resistant from the onset; (4) may have only a minimal EEG (13). At present MELAS cannot be cured. This study retrospectively analyzed clinical data from 34 patients with epilepsy associated with MELAS, and explored their clinical characteristics and prognosis, and analyzed related factors affecting the prognosis.

Males are more common than females among patients with mitochondrial encephalomyopathy, and some studies suggest that being male is a risk factor for MELAS (12, 14). In the present study, the ratio of males to females was 1.43:1 (20:14). It is possible that sex influences the development of MELAS, although the mechanism for this is currently not clear. Therefore, future studies with larger sample sizes are necessary. The age of onset of MELAS is typically between ages 2 and 31 years, and few patients are diagnosed over 40 years of age (15). The average age of the 34 MELAS patients with epileptic seizures in the present study was 22.6 years, and the age range was large (0.5–57 years). This suggests that the possibility of MELAS should be considered for older patients with epilepsy and stroke-like recurrent seizures that cannot be explained by other causes. When epilepsy is the first MELAS symptom and only sign of a disorder, it can be extremely difficult to diagnose patients. In the meantime, only a few patients in our study had slight brain atrophy that was visible on the MRI in the early stages of the disorder. Therefore, we believe that as for some patients, the imaging features of brain atrophy may be the only clue to the early stage of the disease, it is necessary to regularly review their head MRIs for an early diagnosis of MELAS. The main clinical manifestations of MELAS patients include recurrent stroke-like seizures, epilepsy, headache, hearing loss, diabetes, extraocular muscle palsy, and peripheral neuropathy; of these, headache, epilepsy, and stroke-like seizures are considered part of a progressive process and represent different stages of the disorder (16).

During the 2-year follow-up, some observed clinical symptoms improved to varying degrees, including mental symptoms, clumsy speech, vision loss, paresthesia, headache, poor physical activity, and hearing loss. According to literature reports, headaches in MELAS patients could represent both a common neurological manifestation and an epileptic symptom. Some studies indicate that headaches are the only ictal manifestation, particularly in patients with focal epilepsy involving the posterior cortical regions (17).

Seizures frequently occur in mitochondrial diseases like MELAS (6). Our study found that the epilepsy associated with MELAS has various forms and can coexist with multiple types of seizures, which is consistent with previous literature (3). The most common form of epilepsy among patients in the present study was focal epilepsy, of which the consciousness and motor symptoms were most common. The patients with non-motor symptoms of focal epilepsy included two cases of paresthesia (numbness) and one case of mood disorders. Patients with generalized epilepsy all had onset with motor symptoms, most often generalized tonic-clonic seizures. Of the patients, 91.30% had abnormal EEGs, and 8.70% of patients had normal EEGs. It is possible that patients with a normal EEG were at the time of examination, in the compensatory phase after the acute phase of cerebral ischemia and hypoxia, or that the discharge site was deeper. This would result in no obvious abnormal electrical activity being observed. For patients with abnormal EEGs, abnormal waveforms were mainly distributed in the occipital, frontal and temporal lobe. The locations of the abnormal waveforms were roughly consistent with locations of lesions observed in patients' imaging. Slow waves were seen in 56.52% of EEGs; these were mainly focal slow waves and were most common in the occipital area. This could relate to brain atrophy in patients with MELAS or lesions of the cerebral cortex and subcortical white matter. However, we have not confirmed any correlations between EEG characteristics and the prognosis of patients with MELAS and epilepsy.

In our study, the brain MRIs of MELAS patients showed mostly long T2 signals in the cerebral cortex of the temporal, parietal, and occipital lobes, and subcortical white matter, which suggests laminar necrosis of the brain tissue and dynamic changes in the lesions. The lesions were mainly in the temporal and occipital cortex and deep brain nuclei, which are areas of high metabolism and high oxygen consumption. As the course of MELAS progresses, new and old lesions can appear alternately. In some patients, calcification of the basal nucleus, brain atrophy and ventricular enlargement was also observed. In some patients, tall double peaks of lactic acid and decreased NAA peaks were observed by MRS. MRS has an auxiliary role in the diagnosis of MELAS and can detect abnormal metabolism associated with the disease (18). As MELAS progresses, most patients' imaging gradually shows signs of symmetric lesion distribution. This is due to the characteristics of genetic metabolic diseases, which are clearly different from cerebral infarctions and can be used as an important basis for identification.

For treatment, MELAS lacks an effective treatment plan and mainly adopts palliative and symptomatic treatments. Patients can be given large doses of vitamins, coenzyme Q10, adenosine triphosphate, arginine, inosine L-carnitine idebenone, butylphthalide, edaravone and ganglioside, as well as anti-epileptic treatment and other conventional medications (19). It is generally believed that early anti-epileptic treatment is essential for MELAS patients with seizures, but it is worth noting that some anti-epileptic drugs have high mitochondrial toxicity, including valproic acid, carbamazepine, phenytoin and phenobarbital. The use of such drugs should therefore be avoided for patients who might be suffering from MELAS. Anti-epileptic drugs with low mitochondrial toxicity should be used instead. At present, it is believed that levetiracetam can be used as the first-line drug for seizures in patients with mitochondrial encephalomyopathy (20). Many new therapeutic approaches for mitochondrial diseases have also been proposed and are now at different stages of development (21). At 2 years of follow-up, the effective control rate of epilepsy associated with MELAS in the 34 patients was 55.88%. Obviously, there are more measures worthy of implementation to improve the effective control rate of epilepsy.

The statistical analysis of clinical patient data in the complete control, effective, and ineffective groups found a higher incidence of status epilepticus (*P* < 0.001), brain atrophy (*P* = 0.026), and antiepileptic drug use with excessive mitochondrial toxicity (*P* = 0.015) in the ineffective group, compared to the complete control group. The difference between the control group and the effective group symptoms was statistically significant, suggesting a correlation with the poor prognosis of MELAS and epilepsy patients.

The occurrence of MELAS-associated metabolic epilepsy is related to mitochondrial dysfunction (6), high-energy neuron consumption, and a lack of regenerative capacity. Impaired mitochondrial function often affects the central nervous system and can lead to epilepsy and even status epilepticus, which has a serious negative impact on the patient's prognosis (22). The occurrence of status epilepticus can cause neuron death, as well as the possibility of concurrent infection, electrolyte imbalance, acid-base balance disorder, respiratory failure, circulatory failure, and liver and kidney dysfunction, all of which further aggravate neuronal damage in MELAS patients. Status epilepticus causes the patient to die more quickly by seriously damaging the nervous system and even causing permanent epilepsy. Status epilepticus has a high disability and mortality rate (23) and its occurrence often indicates a poor prognosis in patients with epilepsy.

Brain atrophy is the reduction of brain tissue, which can be secondary to enlargement of the ventricles and subarachnoid space. In imaging, brain atrophy can manifest as the diffuse increase, widening and deepening of the sulci; enlargement of the ventricles and cisterns; and widening of the subarachnoid space. Studies have shown that brain atrophy can be used as an objective pathogenesis for patients with epilepsy, and it may even be the only pathogenesis at this time. Brain atrophy can cause epilepsy, or it can occur due to secondary changes in brain tissue in the epileptic brain. Brain atrophy may be related to cortical atrophy, but the specific mechanism for this is not clear. Our statistical analysis suggests that brain atrophy is an indicator of poor patient prognosis, but the mechanisms of brain atrophy and poor prognosis of patients with MELAS and epilepsy remain unclear. Understanding the relationship between brain atrophy and MELAS and the prognosis of patients with epilepsy requires further investigations with expanded sample sizes. We believe that for patients with epilepsy and brain atrophy, in addition to anti-epileptic treatment, neuroprotective drugs should be used early to delay brain atrophy and improve prognosis.

Patients with MELAS and epilepsy should receive anti-epileptic treatment as soon as possible, but some anti-epileptic drugs also affect mitochondria. Therefore, patients with MELAS and epilepsy should instead be prescribed anti-epileptic drugs with low mitochondrial toxicity (24). It is now generally accepted that low-toxicity anti-epileptic drugs should be used in early stages of the disorder, and drugs with high mitochondrial toxicity should be considered only when low mitochondrial toxicity drugs are ineffective or when patients have developed refractory epilepsy. In the present study, 47.06% of patients had used anti-seizure medications with high mitochondrial toxicity (such as sodium valproate, phenobarbital, carbamazepine, etc.). Anti-seizure medications with high mitochondrial toxicity can seriously interfere with antioxidant defense and pathways of the outer respiratory chain and can change the morphology of mitochondria and even accelerate apoptosis (25). Therefore, it is reasonable that the use of anti-seizure medications with high mitochondrial toxicity accelerates the progression of MELAS, leading to poor outcomes.

## Conclusion

In summary, status epilepticus and brain atrophy, along with anti-epileptic drug use leading to high mitochondrial toxicity, are associated with poor prognoses in patients with MELAS-associated epilepsy. Therefore, clinicians and patients should avoid the use of anti-epileptic drugs with high mitochondrial toxicity, control seizures more effectively, and delay the occurrence and progression of brain atrophy where possible, to improve the prognosis of patients with MELAS-associated epilepsy.

Epilepsy associated with MELAS is very common in clinical practice. This summary of the clinical features of epilepsy associated with MELAS and analysis of its prognostic factors can help neurologists increase their understanding of this disorder and improve patient prognoses. At the same time, this research has some shortcomings. This research is a retrospective analysis with a small sample size. The small sample size limited the application of multi-factor analysis. We hope that future studies will expand upon this work with larger sample sizes.

## Data Availability Statement

The raw data supporting the conclusions of this article will be made available by the authors, without undue reservation.

## Ethics Statement

Ethical review and approval was not required for the study on human participants in accordance with the local legislation and institutional requirements. Written informed consent to participate in this study was provided by the participants' legal guardian/next of kin.

## Author Contributions

JL and HM wrote the first draft of this manuscript. WZ and ZC helped revising the manuscript and collected data. ZL and TJ analyzed and interpreted the data, revised the manuscript, and gave final approval. All authors contributed to the article and approved the submitted version.

## Conflict of Interest

The authors declare that the research was conducted in the absence of any commercial or financial relationships that could be construed as a potential conflict of interest.
